# Botany, Nutritional Value, Phytochemical Composition and Biological Activities of Quinoa

**DOI:** 10.3390/plants10112258

**Published:** 2021-10-22

**Authors:** M. Iftikhar Hussain, Muhammad Farooq, Qamar Abbas Syed, Anum Ishaq, Abdullah Ahmed Al-Ghamdi, Ashraf A. Hatamleh

**Affiliations:** 1 Department of Plant Biology & Soil Science, Universidad de Vigo, As Lagoas, Marcosende, s/n, 36310 Vigo, Spain; 2 CITACA, Agri-Food Research and Transfer Cluster, Campus da Auga, Universidad de Vigo, 32004 Ourense, Spain; 3 Department of Plant Sciences, College of Agricultural and Marine Sciences, Sultan Qaboos University, Al-Khoudh 123, Oman; farooqcp@gmail.com; 4 National Institute of Food Science and Technology, Faculty of Food, Nutrition & Home Sciences University of Agriculture, Faisalabad 38000, Pakistan; Qamar.Abbas@uaf.edu.pk; 5 School of Food and Agricultural Sciences, University of Management & Technology, Johar Town, Lahore 54770, Pakistan; anum1797@yahoo.com; 6 Department of Botany and Microbiology, College of Science, King Saud University, Riyadh 11451, Saudi Arabia; abdaalghamdi@ksu.edu.sa (A.A.A.-G.); ahatamleh@ksu.edu.sa (A.A.H.)

**Keywords:** quinoa grains, phytochemicals, anti-oxidants, nutrients, phytosterols

## Abstract

Quinoa is a climate-resilient food grain crop that has gained significant importance in the last few years due to its nutritional composition, phytochemical properties and associated health benefits. Quinoa grain is enriched in amino acids, fiber, minerals, phenolics, saponins, phytosterols and vitamins. Quinoa possesses different human-health promoting biological substances and nutraceutical molecules. This review synthesizes and summarizes recent findings regarding the nutrition and phytochemical properties of quinoa grains and discusses the associated biological mechanisms. Quinoa grains and grain-based supplements are useful in treating different biological disorders of the human body. Quinoa is being promoted as an exceptionally healthy food and a gluten-free super grain. Quinoa could be used as a biomedicine due to the presence of functional compounds that may help to prevent various chronic diseases. Future research needs to explore the nutraceutical and pharmaceutical aspects of quinoa that might help to control different chronic diseases and to promote human health.

## 1. Introduction

The current need of the food industry is the development of innovative and alternative products. However, the sector is saturated due to new products and invasive, foreign low-quality products. Therefore, the use of alternative ingredients compared to those commonly used is the only way to gain new slices of the market. The evolution of the concept of “food” has been profound; it is traditionally understood as the set of food and nutrient elements taken by the body for the normal performance of vital functions. Nonetheless, in addition to satisfying the nutritional and sensory needs, the food should have the capacity to influence the psycho-physical well-being of the organism, preventing the risk of the onset of certain pathologies. In this regard, some pseudo-cereals, such as quinoa, are a potential option. Several biomolecules from quinoa including carotenoids and essential amino acids can be exploited in the manufacturing of food items with a high dietetic–nutritional value.

Quinoa (*Chenopodium quinoa* Willd.) is an annual herbaceous plant. It is considered a pseudo-cereal because the plant belongs to the family of spinach and sugar beets. Quinoa is a native grain-like crop grown originally in the Andean region of South America including Peru, Bolivia, Ecuador, Colombia and Chile. It is a gluten-free food grain crop that is safe for celiac patients [1]. Almost 3000 to 4000 years ago, this wonderful crop was domesticated for human consumption and livestock feed [2]. In recent years, it has been introduced in other regions of the world such as Europe, North America, Australia, China and Japan. Quinoa can adapt well to different environments where the survival of other field crops is extremely difficult. For these reasons, the FAO declared the year 2013 as the International Year of Quinoa.

Quinoa has been the main food of the Inca civilization. In the Quechua language of the Incas, quinoa is called the chisaya mama—“mother of all the seeds”, because, at the time of the Incas and the Aztecs, this food represented, together with the maize and the potato, an essential element of their diets. It was considered a source of life for its beneficial and healing properties and was even venerated as a sacred plant, and was, therefore, known as “Inca gold”. However, following the Spanish conquest, the cultivation of quinoa could not maintain its momentum [3] and is currently limited to circumscribed areas of South America. In recent years, there has been a re-evaluation of quinoa for its agronomic and nutritional characteristics. The possibility of increasing the production of quinoa in other parts of the world, such as in Canada, is being considered and also the possibility of guaranteeing production in terms of quantity and quality to meet the demands of the food industries.

The quinoa plant has a broad genetic diversity that allows it to be highly resistant to cold, salt and drought conditions, with ecotypes growing well at high altitudes and in poor soils, where other cereal crops, such as wheat, rice and maize, do not growth well [4]. Quinoa is a hardy, drought-tolerant plant with a combined precipitation and irrigation requirement of 25–38 cm per years, which is far less than the water requirements of other cereals such as wheat and rice [4]. As long as the soil is naturally moist, plants should not be irrigated until the seedlings show two or three leaves. On the other hand, over-watering during the seedling stages can cause damping off and severe stunting of quinoa, whereas excessive irrigation, after the plant establishment, may result in lodging. In the Andean region, quinoa is usually cultivated in rotation with potato or cereals, without the use of fertilizers or manures. In other countries, quinoa responds well to nitrogen fertilizer; however, over-fertilization may cause excessive vegetative growth leading to lodging [5].

Despite the relatively lower quinoa grain yields ranging from 0.23 t/ha in Mauritania to 7.5 t/ha in Lebanon compared to that of common cereals, quinoa production has intensified quickly in recent years due to the increasing prices in the international market [4]. The price of the quinoa sold by farmers has almost tripled from 2004 to 2012, and is three times the price of soybean and five times the price of wheat [6]. The higher economic profits compared with those from common cereals drive farmers to expand the existing plant scale [7]. High quinoa prices will be sustained (including in China) because the production of quinoa cannot meet the demand in a short-term period from an increasing number of people who intend to include quinoa in their diets [6].

**Figure 1:**
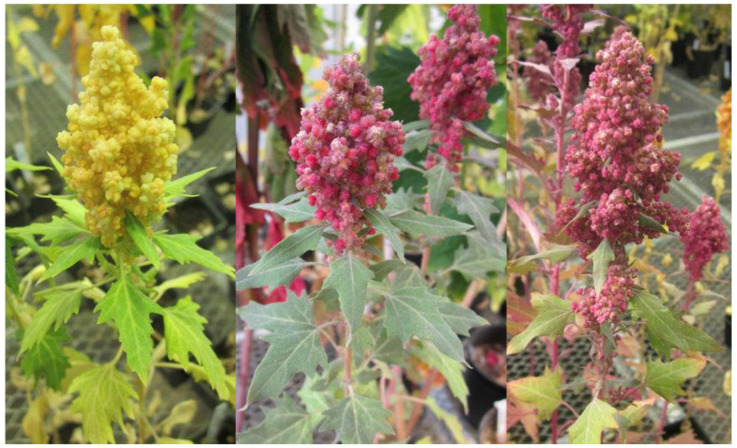
Quinoa plants with two different colors of grains (yellow and purple red).

Quinoa leaves and seeds are edible, with the latter being the principal form for human consumption (Figure 1). Three main storage compartments can be distinguished within the mature quinoa seed (from center to edge): a large central perisperm, a peripheral embryo and a one to two cell layered endosperm only in the micropyle region surrounding the hypocotyl–radicle axis of the embryo [8]. The quinoa endosperm has 1–2 layers; however, the starch is principally stored in the non-living perisperm that occupies around 40% of the volume of the quinoa seed, although small amounts also exist in the embryo but not in the endosperm [8]. The quinoa embryo and endosperm are rich sources of minerals, proteins and lipids [8]. The seeds are round and flattened, about 1.5–4.0 mm in diameter and 0.5 mm in thickness; around 350 seeds weigh one gram, and the seed color ranges from black, white and gray-purple to yellow, red and violet. The varietal identification in quinoa is based on the plant morphology, color of the plant and seeds [2,9]. Quinoa seeds are being used for salad dressings or are cooked similar to rice and are used in soups and/or breakfast cereals. Quinoa use is common in various toasted and baked goods (bread, noodles, cakes, cookies, biscuits, flakes, pancakes and tortillas) [10].

The interest in quinoa stems from its high capacity to adapt to different environments to make it competitive with common cereals. Quinoa can withstand extreme climates such as from sea level to 4000 m above sea level or from 40° South to 2° North. Moreover, it can be cultivated in arid, semi-arid and tropical environments. It is also resistant to frosts and can grow even in drought conditions as well as on cold mountains [11]. Currently there are over 250 varieties of quinoa, the most cultivated varieties include Bear, Cherry Vanilla, Cochabamba, Dave 407, Gossi, Isluga, Kaslala, Kcoito, Linares, Puno, Titicaca, Rainbow, Red lighthouse, Red head and Temuco [12,13].

Quinoa is a rich source of protein, fibers, minerals, vitamins and lipids. Quinoa grains contain essential amino acids and polyphenols [14]. The composition of essential amino acids in quinoa is similar to the amino acid requirement pattern and is higher than that in whole grain and refined wheat. Additionally, quinoa contains a significant amount of minerals. A significant quantity of polyphenols is also present in quinoa seeds [14]. The polyphenols show different pharmacological properties such as anti-allergic, antiviral, anti-inflammatory, cardiovascular protective and anticarcinogenic activity [15].

### Origin and Dissemination

Quinoa originated from the Andean regions such as Peru, Bolivia and Ecuador, where it is still the staple food for the population. Slowly, other foreign crops colonized the most fertile lands, erasing the small terraces occupied by the quinoa and other traditional crops and distorting the landscape; the quinoa plant then ended up being despised by the local population because it was considered food for the poorest, as were the remaining indigenous crops. Ironically, the revaluation of quinoa began abroad, far from its typical areas of cultivation. Nowadays, it is above all thanks to fair trade that quinoa is experiencing a new renaissance, becoming not only a simple product of subsistence for local populations but also an export product. The quinoa of fair trade generally comes from organic farming, and this market is the result of collaboration between the Third World Cooperative Altromercato (CTM), also known as Altromercato, and the ANAPQUI (Asociaciòn Naciònal Productores de Quinoa) of Bolivia. Altromercato is the first association that manages the import of fair-trade products in Italy, and the second in the world, in terms of size and turnover (www.wikipedia.org/altromercato; accessed on 13 April 2019). The association, Altromercato, aims to base its activity exclusively on processes of solidarity, economy and responsible consumption, trying to ensure the suppliers of raw materials a fair price and transparent long-term relationships and also supporting organic farming.

Altromercato opera ANAPQUI (Asociaciòn Naciònal Productores de Quinoa) is an association founded in 1983 in the area of Salar, on the Bolivian plateau. It is a federation of seven regional associations that brings together a total of 5000 small producers of quinoa. The association has the task of buying quinoa from farmers and exporting it to various organizations, including those of fair trade. ANAPQUI’s main aim is to offer producers the opportunity to bring their products to market on more favorable terms, given their inability to provide all the necessary marketing and exportation procedures autonomously. For this purpose, ANAPQUI promotes autonomous forms of organization of producers with a permanent training and information activity. The enhancement of quinoa in the food sector has also been encouraged by the FAO, which has massively promoted the cultivation and diffusion of this pseudo-cereal.

## 2. Quinoa Botany and Morphological Characteristics

Quinoa is a dicotyledonous annual herbaceous plant belonging to the subfamily of the Chenopodiaceae that has some starchy seeds (achenes). Its height ranges from 3 to 7 feet. Depending upon the variety, its woody central stem may be branched or unbranched and varying in color as well (green, red or purple). The flowering panicles appear from the leaf axils along the stem, or they may arise from the top of the plant. Generally, the flowers are self-fertilized though crosspollination may also occur [3].

### 2.1. Leaves

The leaves are made of the petiole and the lamina. The petioles are long, thin and grooved. The length of the petioles varies according to the variety but may vary even within the same plant. The leaf blade is also polymorphic in the same plant and can be diamond-shaped, triangular or lanceolate, flat or wavy [16]. Inside the leaves are calcium oxalate crystals that reduce excessive perspiration allowing the maintenance of adequate humidity inside the plant. The color of the leaves is very variable, from the green in the younger plants to the red or violet with different shades in the more mature plants; they have very pronounced and easily visible veins, deriving from the stem and are generally three in number. There are genotypes with more leaves and others with less; in general, the “Valley” quinoa has abundant foliage, allowing the use of the plant as fodder. In many areas of the Andean region, the young leaves before flowering are considered suitable for human consumption, due to their high nutritional value attributed to their content of vitamins, minerals and proteins.

### 2.2. Root

The plant is deep-rooted, with a highly branched system, which gives it good stability and allows it to withstand drought resistance [17]. The color varies according to the type of soil in which it grows. During the germination, the first part that develops is the radicle, which continues to grow both laterally and vertically reaching up to 180 cm. The root length and the distribution of the lateral roots vary with the genotype and allow a strong anchoring to the ground and a good resistance of the plant to water scarcity. The size of the root is closely related to the height of the plant and can exceed 30 cm, in general; however, it tends to grow deep to form a highly branched system to increase the resistance of the plant to drought. 

### 2.3. Stem

The plant has an erect woody stem, which can be ramified or non-branched, with heights ranging from 30 cm to 3 m, depending on the variety of cultivated quinoa [17] and climatic conditions. It is generally cylindrical, with a resistant bark when the plants are young and a porous one when they mature. The diameter of the stem varies according to the genotype, the sowing distances, the fertilization and the cultivation conditions and ranges from 1 to 8 cm. The ramifications of the configuration of the plant can be modified by insect attack, mechanical damage or by some cultural practices. As genotypes, seeding density, nutrient availability and growth areas vary, the plants may be widely branched (“Sea-level” quinoa), without branching (plateau) or intermediately branched.

The color of the stem, similar to that in amaranth, may vary from pale yellow to red, depending upon the genotypes and the phenological phases [18]. The color often shows striations, with the leaf axils and branches being red or purple. The stem has a cutinized epidermis, with membranes of compact cellulose. Within it, the stem contains a marrow that disappears upon ripening, leaving the stem dry and empty. This stem, owing to high pectin and cellulose contents, can be used in the manufacturing of paper and cardboard. Wide leaves, attached to the stem alternating on four levels, are characterized by polymorphism. The uppermost leaves are small and lanceolate, while those at the bottom are large, rhomboidal or triangular [19].

### 2.4. Inflorescences and Flowers

The quinoa inflorescence is a typical panicle, consisting of a central axis and secondary and tertiary branches with the pedicels holding the glomeruli. The panicle may have sparse (amarantiforme) or compact (glomerular) inflorescences. There are also forms that present transitional characteristics between the two groups [17]. An inflorescence in compact groups with close pedicels is called glomerular, where the inflorescence with elongated glomeruli and several secondary and tertiary branches attached to central axis with flowers grouped in fairly loose tufts is termed as amarantiforme. This inflorescence type has been named so due to its resemblance to the inflorescence of the genus *Amaranthus*.

The length of the panicle is variable, depending on the genotype, the type of quinoa and the fertility conditions of the soil, and ranges from 30 to 80 cm in length and from 5 to 30 cm in diameter. The number of glomeruli per panicle varies from 80 to 120 and the number of seeds per panicle can range from 100 to 3000. The large panicles may yield up to 500 g of seed per inflorescence.

The plants produce panicle inflorescences having hermaphrodite and pistillate flowers. The hermaphrodite flower possesses five imbricate sepals and five antisepalous stamens; its filaments extend laterally forming a ring of nectariferous tissue surrounding the ovary having modified stomata. The gynoecium is bicarpellate with unilocular ovary having a single campylotropous ovule [17]. Quinoa plants are also hermaphrodites, which mean they are characterized by self-pollination [20]; however, cross-pollination (10–15%) can also take place [21]. The hermaphroditic flowers are located at the distal end, while the female flowers are in the proximal end [22]. They are self-fertile, and pollination is generally anemophilous (through the wind). Upon ripening, the plant produces a panicle containing seeds, called an achene, which resemble those of millet in appearance.

### 2.5. Fruit

The fruit is an indehiscent achene, with a spherical, conical or ellipsoidal shape, with a diameter varying between 1.0 and 2.6 mm. The coloring of the pericarp varies extremely from yellowish to gray with magenta. It is held with five lobes of the perianth, which are easily removable by abrasion. The fruits are small in size, and the weight of 1000 achenes ranges from 1.9 to 4.3 g [23]. The seeds have three major components viz. episperm, embryo and perisperm. A thin layer called the pericarp surrounds the achene. The pericarp has a rough and brittle outer surface that can be easily removed upon rubbing. This part contains the saponins, which give a bitter taste to the seed. The episperm is located below the pericarp.

The embryo is made up of two cotyledons and constitutes 30% of the total volume of the seed, surrounding the perisperm like a ring, with a curvature of 320°. Compared to other seeds, the embryo contains 35–40% of the total seed proteins, while the perisperm presents only 6.3–8.3% of the total proteins [8]. The perisperm is whitish in color and acts as the main storage fabric and consists mainly of starch granules. This represents almost 60% of the seed surface. The quinoa endosperm is composed of several layers around the embryo. After seed hydration, endosperm cells come into contact with the embryo and are rapidly consumed during its growth.

## 3. Nutritional Profile

Quinoa is pseudo-cereal rich in proteins, lipid, fiber, vitamins and minerals [24]. Quinoa grains are a good source of natural compounds with anti-oxidant activities and biological properties [25]. However, the composition of different genotypes varies significantly [26]. Chen et al. [27] classified quinoa into two groups. Group A containing higher phytochemicals and polyunsaturated fatty acids and group B containing higher linolenic and long-chain fatty acids. Both groups can be utilized in food products. Quinoa grain has an excellent nutritional profile, starch (32–60%), protein (10–18%) and fat (4.4% to 8.8%), while the ashes, formed mainly from potassium and phosphorus, constitute 2.4% to 3.7% and the fiber ranges from 1.1% to 13.4% [28,29]. The quinoa grains also contain a fair amount of vitamin B and vitamin E, a fat-soluble anti-oxidant vitamin.

The use of quinoa grains in food requires a series of operations aimed at the removal of fractions rich in antinutritional compounds, in particular saponins, located in the perianth and the pericarp, which are responsible for the sensation of bitterness and have negative effects on the intestinal mucosa. To remove the saponins, the quinoa grains are washed with tap water followed by hand rubbing and drying. However, this process may require several cycles [29,30,31,32]. The drying step is carried out under controlled conditions to avoid modifications of the quinoa seed constituents (such as starch gelatinization and protein denaturation), which may lead to significant changes in the nutritional and rheological properties of the quinoa flour. The summary of the impact of quinoa products on human health is given in Table 1.

### 3.1. Protein

The quinoa grain is an important source of dietary proteins [31]. According to the reports of Abugoch [32], the proteins present in quinoa grains include albumins (35%) and globulins (37%) and a lower percentage of prolamins. The quality of proteins present in quinoa is comparable to milk protein (casein). Quinoa proteins constitute all essential amino acids such as tryptophan, histidine, isoleucine, leucine, lysine, methionine, phenylalanine, threonine, tryptophan, tyrosine and valine) [2,32]; this is why, this is considered a complete food [1].

The intake of proteins is an issue for the populations who rarely consume animal proteins, and therefore, their diets should include proteins of plant origin. Quinoa-based products are an apt option in this regard, because unlike other conventional cereals, quinoa does not suffer significant losses in protein content during industrial processing. Moreover, the amino acid composition of the quinoa proteins makes the organic value of quinoa superior to that of other traditional cereals. Electrophoretic studies showed that quinoa proteins consist of two large fractions—11S-globulin and 2S-protein. 11S-globulin, also called chenopodium [39,40], and represents about 37% of the total protein. This fraction contains polypeptides with a molecular weight of 22–23 and 32–39 kDa and has a relatively low content of amino acids (methionine and cysteine) [40]. The 2S-protein fraction has a molecular weight of 9 kDa and has a high content in cysteine, arginine and histidine, but it is relatively poor in methionine [39].

Quinoa has more than twice the lysine content of wheat, maize and rice. The lysine content in quinoa grains is equal to 96% of the quantity sufficient to reach the FAO standards and, therefore, remains a limiting amino acid. However, this is the highest value ever among all cereals. Leucine is the second limiting amino acid, but even in this case, it is a moderate limitation. According to the FAO standards, the content of this amino acid is 91% of the optimal quantity. Several traditional and modern methods are employed for protein isolation and characterization. This may include wet fractionation and dry fractionation methods. The first one involves huge consumption of water, chemicals and energy [41]. As compared to wet fractionation, dry fractionation was more efficient because it keeps the nutritional properties intact [42]. Motta et al. [43] reported the glutamic acid contents in steamed (2.0 g/100 g) and boiled (2.1 mg/100 g) quinoa seeds. Histidine and aromatic amino acids have also been reported in significant quantities.

### 3.2. Lipids

Quinoa was considered an alternative to oil seeds due to its lipid composition. The fat content of quinoa is quite high (5–10%) compared to that in common cereals and is mainly localized in the embryo [8]. The polar lipids represent about 25% of the total lipids consisting mainly of phospholipids (lysophosphatidyl ethanolamine and choline). Quinoa oil possesses high anti-oxidant activity, high contents of polyunsaturated fatty acids (63% of total) and a significant amount of tocopherols (2.5 mg/g of oil) [44]. Of the neutral lipids instead (glycerides and sterols), triglycerides account for 74% and diglycerides for 20%, while monoglycerides and waxes represent 3%, respectively. Several fatty acids such as linoleic acid (18:2n-6), linolenic acid (20:3n-6) (55–60%) and oleic acid (18:1 *cis*-9) (30%) constitute the main composition of fatty acids. Quinoa seed is also the main source of different essential fatty acids including omega-6 and omega-3 fatty acids. Linoleic acid is metabolized to arachidonic acid and linolenic acid to eicosapentaenoic acid (EPA) and docosahexaenoic acid (DHA) [45]. EPA and DHA play important roles in the metabolism of prostaglandins, thrombosis and atherosclerosis.

Quinoa seed exhibits a significant amount of oil that contains monounsaturated (oleic) and polyunsaturated (linoleic and linolenic) fatty acids. The oil is particularly stable due to the presence of a high content of natural anti-oxidants, such as α-tocopherols (69–75 mg/100 g of oil) and γ-tocopherols (76–93 mg/100 g of oil). The content of these compounds decreases up to 45 and 23 mg, respectively, after oil refining [23]. The quinoa grain also has high quality and quantity of oil due to which it has been defined by some authors as an oily pseudo-seed [23]. The high level of unsaturation may significantly increase the susceptibility of the lipid fraction to oxidative rancidity, but the presence of natural anti-oxidants especially tocopherols limit the oxidation initiation by acting as a natural defense against it [10]. Quinoa contains different phytosterols especially squalene that has shown anti-oxidant activity and helps to maintain cardiovascular health and to cure tumors in human beings. Quinoa seed showed a significant portion of phytosterols including b-sitosterol that possess functions to decrease the low-density lipoproteins [45]. Meanwhile, several other biological properties of phytosterols including anti-inflammatory, anti-oxidant and antitumor activity and cholesterol reduction have also been reported [46].

### 3.3. Carbohydrates

Based on the degree of polarization, carbohydrates can be grouped into simple sugars (monosaccharides, disaccharides), oligosaccharides and polysaccharides (starches). Carbohydrates play a vital role in nutrition and have different effects on metabolic processes such as diabetes, blood glucose, protein glycosylation. Quinoa seed has the characteristic of lowering hypoglycemic effects and decreasing free fatty acids and, thus, serves to exert a food nutraceutical impact through its carbohydrates. As compared to pasta and gluten-free bread, the glycemic index is lower and, hence, significantly reduces the free fatty acids.

According to reports of Jancurová et al. [47], quinoa has a significant amount of carbohydrates that are in the range of 67–74% of total dry matter, and the amylose contents are near 11%. Starch granules are lower than those reported for maize (range 1–2 μm) or wheat (2–40 μm). Meanwhile, some other carbohydrates are also documented, which include monosaccharides (2%) and disaccharides (2.3%), crude fiber (2.5–3.9%) and pentosans (2.9–3.6%) [48]. The small granule diameter of quinoa starch is useful for enhancing binding and reducing breakability [49]. Several researchers reported that quinoa is a good thickener for soups, sauces and flours. The gelling point of quinoa is sufficiently less and is more durable at low storage temperatures. A smooth texture and creamy nature can be obtained from quinoa seeds [2]. The carbohydrate contents of some quinoa genotypes are reported in Table 2. Sucrose is present in significant quantities compared to other sugars [50]. Despite this apparent difference, it can be said that the sugar content in quinoa is very similar to that of amaranth [51].

### 3.4. Starch

Starch is the key constituent in the quinoa seed and constitutes up to 70% of seed biomass. In starch, several polysaccharides joint together along with several glucose subunits. These molecules are mostly joined together through glycoside bonds. It is composed of two polymers: amylose, which is a linear polymer in which the glucose units are linked together with α (1 → 4), and amylopectin bonds, which is a branched polymer that has basic structure chains similar to amylose that arrange themselves to form a branched structure through the insertion of side chains through α bonds (1 → 6). Several studies have been undertaken to characterize quinoa starch [52,53]. The granules are located in the perisperm, singly or aggregated to form more complex structures, formed by hundreds of individual granules [54]. The starch in quinoa is highly branched, with a minimum polymerization of 4600 units of glucan and a maximum of 161,000 units. The length of the chain may depend on the variety of quinoa but is in the order of 500–6000 glucose units. The average degree of polymerization of quinoa amylose (900) is lower than that of barley (1700). Amylose has an average of 11.6 chains per molecule; the length of the quinoa amylopectin chain is about 6700 glucan units. Quinoa amylopectin consists of short chains (8 to 12 units) and longer chains (13–20 units), compared to the other starches of the endosperm cereals. Quinoa starch has low digestibility and extractability due to protein binding [55].

Several studies documented the presence of amylose in quinoa, ranging from 7% to 27%, which is much higher compared to that in barley and rice [56]. The starch gelatinization properties indirectly depend upon different factors that include the size, the proportion and the type of crystalline structure and the ultrastructure of the starch granules. Quinoa starch gelatinizes at relatively low temperatures, similar to those for the gelatinization of wheat and potato starch, but lower than the gelatinization temperature of other large granules.

### 3.5. Fiber

Quinoa is generally considered an important source of fiber. The quinoa washing and abrasion processes to eliminate saponins do not significantly influence the fiber content [57]. According to some reports [58], dietary fiber contents in quinoa are equal to those of cereal and legume grains, but their amount is less than buckwheat. According to research conducted on animal models, quinoa grains exhibit arabinans and rhamnogalacturonan-I and have shown stomach metabolic disorders in rats [59]. Although, the fiber composition of quinoa is different from that of other cereal grains, the biochemical and therapeutic potential of quinoa should be studied to understand its specific physiological impact.

### 3.6. Minerals

The outer layers of the pericarp possess several minerals such as potassium, calcium, magnesium and phosphorus [29,52]. Based on the recommended daily dose (RDA), quinoa is a good source of magnesium, phosphorus and iron. In this context, the iron content (8–9 mg/100 g) is higher than that in common cereals; however, the presence of antinutritional compounds such as saponins and phytic acid can significantly reduce the bioavailability of some minerals [60]. Different treatments such as decortication and ash washing reduce the mineral content of 12–15% for iron, zinc and potassium, while it is 27% and 3% for copper and magnesium, respectively.

### 3.7. Vitamins

Quinoa is a good source of vitamins E and B and folic acid, which are concentrated in the embryo [61,62]. Quinoa possesses a significant amount of vitamin C, riboflavin, folic acid and thiamine compared to other cereals. The dietary requirements of an adult can be met through the consumption of 100 g of quinoa, which may provide the required pyridoxine and folic acid levels. Consumption of 100 g of quinoa grains may fulfill 80% of the DR of children and 40% of the protease, cellulase and hemicellulase requirements [63]. Mechanical abrasion increases the α-amylase and protease activity [63]. In contrast, the levels of cellulase and hemicellulase decrease with abrasion, thus suggesting the presence of the same in the pericarp.

## 4. Secondary Metabolites

### 4.1. Phytoecdysteroids

Among the secondary metabolites, phytoecdysteroids defend plants from insect pests and nematodes [64]. Phytoecdysteroids are polyhydroxylated compounds having a cyclopentanoperhydrophenanthrene ring. However, phytoecdysteroids present in the plants have a quite diverse structural configuration [65]. Phytoecdysteroids are mostly present in the bran portion of the grain as free and polar/non-polar conjugated compounds and are classified based on the number of carbon atoms in the structure as C27- and C28-phytoecdysteroids [66]. Quinoa is the only pseudo-cereal that possesses a significant quantity of phytoecdysteroids ranging from 138 to 570 µg/g [67]. The quinoa plant contains about 36 different types of phytoecdysteroids [68], out of which, C-27 phytoecdysteroids, which have several health benefits [68], are found in the highest concentration [45]. For instance, phytoecdysteroids possess anti-aging properties and are beneficial to prevent skin aging because of their anti-oxidant potential due to their metal ion chelating ability and free radical scavenging activity [69]. Moreover, phytoecdysteroids have also been used as a safe and effective replacement for anabolic steroids [70]. Additionally, these bioactive compounds are quite helpful in boosting the development of skeletal muscles and, thus, play an important role in increasing physical performance [71]. Likewise, several in vivo investigations have confirmed the role of quinoa phytoecdysteroids in preventing the problem of obesity. In a study, the supplementation of quinoa extract to mice fed with a high-fat diet was reported to be beneficial in controlling obesity [72]. The reduction in fat mass due to the dietary administration of quinoa is mainly due to elevated oxidation of carbohydrates and fecal defecation of lipids. Furthermore, the potential of quinoa phytoecdysteroids in preventing diabetes through reduced oxidative degeneration and improved blood glucose transportation has also been reported [73].

### 4.2. Saponins

Saponins are bioactive compounds present in the pericarp of quinoa grains that impart a bitter taste. Saponins are chemically composed of a triterpenoid or steroidal aglycone with one or more sugar moieties such as glucose, galactose, arabinose, xylose and glucuronic acid [74]. Saponins present in the quinoa grains are highly diverse in their structural characteristics, and studies reveal that about 68 different types of saponin compounds have been identified in crude quinoa seed extract by using nano-HPLC electrospray ionization multistage tandem mass spectrometry (nLC-ESI-MS/MS) [75]. The amount of saponins present in quinoa grains depends on its cultivar and can be classified into ‘sweet’ (<0.11% of saponins) or ‘bitter’ (>0.11% saponins) [76]. Meanwhile, bitter-flavored varieties of quinoa contain higher saponin concentrations as compared to sweet varieties [48]. Saponins are beneficial for crop protection against insect/bird herbivore and microbial infection, which accelerates the organic production of this crop. Saponins also possess several health-promoting impacts due to their vast pharmacological functionalities. For instance, dietary incorporation of saponins helps to lower the blood cholesterol levels due to their hemolytic action after directly interacting with the blood cells [77]. Additionally, saponins do not impart any toxic or harmful effect on human health after ingestion. However, saponin concentrations should be lowered during the processing of quinoa seeds as they impose an inhibitory effect on the digestibility and bioavailability of quinoa proteins. Saponin concentrations can be reduced by treating the quinoa seeds with cold, alkaline water followed by mechanical abrasion [78]. Moreover, several breeding strategies have also been adopted to develop new varieties of quinoa with lower saponin contents (<0.11% free saponins). The key functional aspects of quinoa saponins in improving human health include antiviral activity, antifungal ability, anticancer properties, antithrombotic effects, hypocholesterolemic potentiality, diuretic potential, hypoglycemic action and anti-inflammatory characteristics [2,75]. The antifungal effect of quinoa’s saponins has been reported against *Candida albicans* [79]. The anti-inflammatory action of saponins is mainly attributed to the 3-O-β-d-glucopyranosyl oleanolic acid. Quinoa saponins decrease the levels of the inflammatory mediators and inhibit the release of inflammatory cytokines including interleukin-6 (IL-6) and tumor necrosis factor- (TNF-) and in lipopolysaccharide-induced RAW264.7 cells [46]. Due to their surfactant property, saponins can boost drug absorption through mucosal membranes. The property of insoluble complex formation of some saponins with minerals such as zinc and iron decrease the absorption and bioavailability of these minerals in the gut [47].

### 4.3. Phenolic Compounds

Phenolics consist of a large and diverse class of compounds comprising the hydroxyl group(s) attached to at least one aromatic hydrocarbon ring. Phenolics have high structural stability, which determines the strong anti-oxidant potential of these compounds [80]. Quinoa grains contain free phenolic compounds in the range of 167.2–308.3 mg gallic acid equivalents per 100 g of dry weight [81,82]. The range of a free fraction of total phenolic contents in seven varieties of quinoa was determined to be from 53.5% to 78.0%, among which ferulic acid and gallic acid were prominent compounds. Flavonoids in quinoa seeds include quercetin, rutin and kaempferol derivatives. The quinoa grains also possess bound phenolics, which are so named because they are bound with structures of the cell wall such as pectin, lignin cellulose, hemicellulose (arabinoxylans) and also with rod-shaped structural proteins [83,84,85,86,87,88,89,90,91,92,93,94,95,96,97,98]. Quinoa grains contain a lower amount of bound phenolics as compared to the free phenolics [14,81,85]. Bound phenolic compounds are mostly present in the leaves of quinoa but not in the seeds [84].

The phenolic compounds present in different varieties of quinoa play a vital role in preventing the problem of diabetes and obesity [81]. Over the last two decades, phenolic compounds have gained much interest due to their chronic-disease-prevention ability and health benefits [82]. Dietary phenolics help in maintaining gut health by regulating the microbial balance of the gut. Individual phenolic acids contribute to improving the metabolism and cell signaling and, thus, exert significant anticancer, anti-inflammatory, anti-obesity, antidiabetic and cardioprotective effects [80,83].

#### 4.3.1. Phenolic Acids

**Figure 2:**
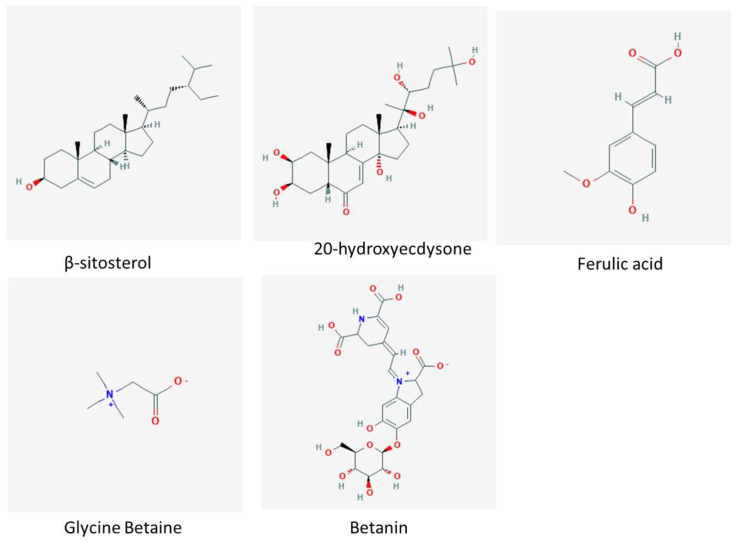
Secondary metabolites and bioactive molecules present in the quinoa grains.

There are free and chemically bound phenolic acids in the cell wall of quinoa leaves [84,85]. Quinoa leaves and seeds contain different types of phenolic acids and their derivatives hydroxycinnamic acid and hydroxybenzoic acid, which possess considerable health-promoting activities such as antihypertensive, anti-oxidative, antidiabetic, anti-inflammatory and anticarcinogenic effects. The concentration of phenolic acids in quinoa seeds varies from one to other variety [98,99,100,101,102,103,104] (Table 3 and Figure 2).

#### 4.3.2. Flavonoids

The quinoa plant has a vast variety of flavonoids including benzoic acid, vanillic acid, syringic acid, p-coumaric acid and ferulic acid [105]. Flavonoids are considered the second most abundantly present components in the quinoa seeds [81] (Table 4).

##### 4.3.2.1. Flavonol Glycosides

The flavonol glycosides are the most abundant flavonoids in the quinoa leaves and seeds [85]. Quinoa contains 12 different types of flavonol glycosides comprised of kaempferol and quercetin derivatives, with an average individual concentration of 839 μg/g on a dry weight basis [86]. 

##### 4.3.2.2. Isoflavones

Plant-based isoflavones showed significant beneficial health impact to humans. Isoflavones were firstly identified in quinoa plants by Lutz et al. [87], whom confirmed the presence of daidzein in the range of 0.70 to 2.05 mg/100 g and genistein from 0.05–0.41 mg/100 g among seeds of 10 distinct varieties of quinoa. 

### 4.4. Betalains

Quinoa contains betalain, which is a water-soluble phytochemical that acts as a natural anti-oxidant and, thus, helps in the prevention of cancer [88]. The red, black and yellow colors of quinoa seeds and their vegetative parts are due to betalain [10]. Betalain pigment contains nitrogen aromatic indole compounds derived from tyrosine (betacyanins that are red-violet and betaxanthins that are orange-red in color) [14]. Betalain is present in different varieties of quinoa in a diversified range. The betalain content (sum of betaxanthins and betacyanins) in some quinoa seeds ranges between 0.15 and 6.10 mg/100 g [89], while other varieties do not even contain detectable levels of betalain [90]. Betanin and isobetanin are most prominently present in the quinoa seeds and have similar health promoting activities such as anti-oxidant and antimicrobial and anti-inflammatory activity [14]. However, as compared to polyphenols, betalain shows higher anti-oxidant activity [91]. Betalain is used as an ingredient in functional foods due to its anticancer, antimicrobial, antilipidemic and anti-oxidant activities [92]. Microencapsulation has been studied recently to stabilize these compounds [93]. Maltodextrin microencapsulations having low saponins and high betacyanins show unique health-promoting activities. Betalain can be used as a natural dye due to its stability at pH 3–7. The United States Food and Drug Authority (U.S. FDA) and the European Union have approved betalain as a natural colorant with an E-number (E-162) in soups, sauce, dairy products pharmaceuticals and cosmetics [94]. Quinoa seed hulls are rich in betalain, which in combination with saponins produces a highly beneficial ingredient for food and pharmaceutical industries [95].

## 5. Conclusions

Quinoa is an important grain crop with excellent nutritive characteristics and phytochemical composition. Gluten-free quinoa grains are a valuable source of energy for several actors of society including children, the elderly and high-performance athletes. Quinoa has been a unique source of nutrition and food supplement to thousands of malnourished humans globally. Current knowledge regarding the use of quinoa and its human health benefits should be enhanced among all players and actors of society. Quinoa provides important fat-soluble anti-oxidant vitamins (vitamin group B and vitamin E). The availability and concentration of many minerals in quinoa are higher compared to those in other cereals. However, antinutritional compounds (saponins) should be removed to avoid the sensation of bitterness. Future research should include more and comprehensive clinical trials to gain more knowledge and understanding regarding possible action mechanisms of phenolics and flavonoids in human metabolic disorders and diseases.

## Figures and Tables

**Table 1:** The effect of quinoa products in human health.

Study Participants	Treatment	Duration	Outcomes	Conclusions	References
50–65-month-old boys	100 g quinoa-added to baby food/day	15 days	Increase in plasma level of insulin-like growth factor-1	Potential role in reducing childhood malnutrition	[33]
18–45-year-old students	Quinoa cereal bars	30 days	Decrease in total cholesterol, triglycerides and low-density lipoprotein levels	Potential role in preventing cardiovascular disease	[34]
Post-menopausal, overweight women	25 g quinoa flakes and cornflakes/day	4 weeks	Reduction of total cholesterol, triglycerides and low-density lipoprotein levels and thiobarbituric acid reactive substances Increase of urinary secretion of enterolignans	Beneficial effect on metabolic parameter modulation	[35]
Celiac patients	50 g quinoa/day	6 weeks	Improved histological and serological parameters Mild hypocholesterolemic effect	Quinoa consumption is safe for celiac individuals	[36]
Overweight and obese participants	50 g quinoa/day	12 weeks	Reduction of serum triglyceride level Reduction of metabolic syndrome	Potential role in preventing obesity	[37]
35–70-year-old healthy, overweight males	Quinoa-enriched bread (with 20 g quinoa flour)/day	4 weeks	Reduction in blood glucose and low-density lipoprotein levels	Potential role in preventing cardiovascular disease	[38]

**Table 2:** Proximate composition of quinoa grains (g/100 g) [47, 48, 49, 50].

Quinoa Genotypes	Moisture	Proteins	Fat	Crude Fiber	Ash	Carbohydrates
Ccoito	8.47 ± 0.08	14.72 ± 0.11	5.33 ± 0.06	1.81 ± 0.02	2.83 ± 0.00	68.1
INIA-415 Pasankalla	9.76 ± 0.07	12.69 ± 0.06	6.85 ± 0.10	2.2 ± 0.02	2.49 ± 0.03	67
Roja de Copotaque	8.3 ± 0.07	11.51 ± 0.10	5.22 ± 0.08	2.26 ± 0.02	2.93 ± 0.05	70.8
Witulla	8.81 ± 0.08	12.28 ± 0.00	5.32 ± 0.01	2.62 ± 0.02	2.57 ± 0.04	69.5
03-21-0093	8.47 ± 0.07	11.79 ± 0.11	nd	Nd	2.76 ± 0.02	nd
Salcedo INIA	8.26 ± 0.05	13.23 ± 0.01	5.3 ± 0.09	5.3 ± 0.2	2.37 ± 0.05	70
Commercial 1	10.13 ± 0.05	13.18 ± 0.01	6.51 ± 0.04	6.51 ± 0.03	2.34 ± 0.10	63.6
Commercial 2	11.51 ± 0.04	13.48 ± 0.06	6.34 ± 0.07	6.34 ± 0.03	2.27 ± 0.10	59.4
Huaripongo	10.34 ± 0.02	11.32 ± 0.01	6.14 ± 0.01	6.14 ± 0.01	2.92 ± 0.04	67.8
03-21-1181	9.37 ± 0.06	11.89 ± 0.02	3.95 ± 0.03	3.95 ± 0.01	3.12 ± 0.02	69.8
Mean ± SD	9.34 ± 1.1 ^a^	12.61 ± 1.1 ^a^	5.66 ± 0.09 ^a^	5.66 ± 1.7 ^a^	2.66 ± 0.3 ^a^	67.3 ± 3.7 ^a^

Means within a column with the same superscript letter are not significantly different (*p* < 0.05).

**Table 3:** Secondary metabolites identified from different quinoa varieties.

Variety	Observed Compounds/Reports	References
Minttumatilda	Vanillic acid, gallic acid, p-benzoic acid, syringaldehyde, ferulic acid	[99]
Four varieties of white (Q-w1), red (Q-r1 and Q-r2) and black (Q-b1) and three varieties of white (Q-w2), red (Q-r3) and black (Q-b2)	Gallic acid, caffeic acid, ferulic acid, p-coumaric acid, p-OH benzoic acid, vanillic acid, protocatechuic acid	[99]
Q25, Q50, Q100, Q25S, Q50S, Q100S	1-O-galloyl-β-d-glucose, acacetin/questin/apigenin-7-methyl ether, protocatechuic acid 4-O-glucoside, vanillic glucoside, penstebiosided, canthoside A/2-hydroxybenzoate 2-O-β-d-apiofuranosyl-(1→6)-O-β-Dglucopyranoside, ferulic acid 4-O-glucoside, ethyl-m-digallate, (epi) gallocatechin, quercetin 3-O-(2,6-di-α-l-rhamnopyranosyl)-β-d-galactopyranoside, kaempferol 3-O-β-d-apiofuranosyl(1‴→2″-O-[α-l-rhamnopyranosyl(1‴→6″]-β-d-galactopyranoside, kaempferol 3-O-β-d-apiofuranosyl(1‴→2″-O-[α-l-rhamnopyranosyl(1‴→ 6″]-β-d-galactopyranoside isomer, kaempferol 3-O-(2,6-di-α-l-rhamnopyranosyl)-β-d-galactopyranoside (mauritianin), quercetin 3-O-[ β-d-apiofuranosyl(1‴→2″)]-β-d-galactopyranoside, rutin, quercetin glucuronide, quercetin 3-O-glucoside	[85]
Red quinoa (RQ), white quinoa (WQ), Mengli 1 (gray quinoa, GQ), Altiplano, djulis cultivar	Protocatechuic acid, p-hydroxybenzoic acid, vanillic acid, caffeic acid, syringic acid, p-coumaric acid, ferulic acid, isoferulic acid	[100]
White and red quinoa	Gentisic acid, L-alpha-hydroxy isovaleric acid, 3-hydroxybenzoic acid, p-coumaric acid, methyl-b-d-galactopyranoside, 3-(3,4-dihydroxyphenyl)propionic acid hesperidin	[100]
Puno, Titicaca	Ferulic acid, 5-O-caffeoylquinic acid, gentisic acid, p-coumaric acid, ellagic acid, pterostilbene, coniferyl aldehyde	[13]
White quinoa, red quinoa, black quinoa	Protocatechuic acid, p-hydroxybenzoic acid, vanillic acid, syringic acid, p-coumaric acid, ferulic acid, sinapic acid, isoferulic acid	[6]

**Table 4:** Secondary metabolites (flavonoid) identified from different quinoa varieties.

References		Flavonoids
[101]		Quercetin 3-O-(2,6-di-α-Lrhamnopyranosyl)-β-Dgalactopyranoside
[85]		Quercetin 3-O-(2,6-di-α-Lrhamnopyranosyl)-β-Dgalactopyranoside
[85]		Quercetin-3-O-(2″-apiosyl)-rutinoside
[85]		Quercetin glucuronide
[101,102]		Kaempferol 3-O-(2,6-di-α-Lrhamnopyranosyl)-β-Dgalactopyranoside
[101,102]		Kaempferol 3-O-(β-d-apiofuranosyl (1″→2″)-α-l-rhamnopyranosyl) β- Dgalactopyranoside
[101]		Kaempferol 3-O-[β-d-apiofuranosyl (1″→2″)] β-d-galactopyranoside
[101]		Kaempferol-3-O-(α-Lrhamnopyranosyl) -β- Dgalactopyranoside, kaempferol glucuronide
[14]		Kaempferol 3-O-glucoside, kaempferol pentosyl rhamnoside, kaempferol
[13]	Puno, Titicaca	Quercetin, isorhamnetin, quercetin−3-Ogalactoside, isorhamnetin−3-Orutinoside, rutin, naringin, aesculin, phlorizin, eriodictyol
[103]	BRS Piabiru	Quercetin, kaempferol 3-O-rutinoside, quercetin 3-O-(2″,6″-di-O-α-l-rhamnoside)-β-d-galactoside, quercetin 3-O-(2″-O-β-apioside-6″-O-α-rhamnoside)-β-galactoside, kaempferol 3-O-(2″,6″-di-O-α-rhamnoside)-β-galactoside, kaempferol 3-O-(2″,6″-di-O-α-rhamnoside)-β-glucoside

## Data Availability

This is a review article. All the data is presented in the manuscript.
